# Nervous system-wide profiling of presynaptic mRNAs reveals regulators of associative memory

**DOI:** 10.1038/s41598-019-56908-8

**Published:** 2019-12-30

**Authors:** Rachel N. Arey, Rachel Kaletsky, Coleen T. Murphy

**Affiliations:** 1 Department of Molecular and Cellular Biology and Center for Precision Environmental Health, Baylor College of Medicine, Houston, TX 77030 USA; 2 Department of Molecular Biology & LSI Genomics, Princeton University, Princeton, NJ 08544 USA

**Keywords:** Learning and memory, Genomic analysis

## Abstract

Presynaptic protein synthesis is important in the adult central nervous system; however, the nervous system-wide set of mRNAs localized to presynaptic areas has yet to be identified in any organism. Here we differentially labeled somatic and synaptic compartments in adult *C. elegans* with fluorescent proteins, and isolated synaptic and somatic regions from the same population of animals. We used this technique to determine the nervous system-wide presynaptic transcriptome by deep sequencing. Analysis of the synaptic transcriptome reveals that synaptic transcripts are predicted to have specialized functions in neurons. Differential expression analysis identified 542 genes enriched in synaptic regions relative to somatic regions, with synaptic functions conserved in higher organisms. We find that mRNAs for *pumilio* RNA-binding proteins are abundant in synaptic regions, which we confirmed through high-sensitivity *in situ* hybridization. Presynaptic PUMILIOs regulate associative memory. Our approach enables the identification of new mechanisms that regulate synaptic function and behavior.

## Introduction

Neurons are polarized, structurally complex cells comprised of functionally distinct compartments, with dendrites, somatic regions, and axon terminals often operating in different microenvironments^1^. These compartments must often rapidly respond to and integrate discrete, spatially restricted stimuli. One of the mechanisms by which synapses coordinate dynamic responses is through localized protein synthesis in synapses, as protein transport alone from the soma is too slow to meet timing demands of synaptic signaling. It was long thought that local translation in the adult brain exclusively occurred in postsynaptic, not presynaptic, compartments, due to the failure of electron microscopy studies to visualize polysomes in presynaptic terminals^2^, though recent expansion microscopy techniques have found that ribosomes are indeed present in presynaptic terminals^3^.

The importance of dendritic local translation in neuronal plasticity was first identified when it was observed that protein-synthesis-dependent long-term potentiation (LTP) and long-term depression (LTD) occur when the soma is physically disconnected from postsynaptic regions^4–6^. Dendritic local translation is also thought to play an important role in memory storage, as it provides a potential mechanism for the strengthening of specific synapses. There have been many recent advances in characterizing mRNAs localized to dendritic regions, including the identification of over 2000 synaptic mRNAs localized to the neuropil of the hippocampus^7^ as well as compartment specific 3′ UTR usage^8^, and the development of new tools to study and visualize the translation and localization of specific mRNAs (reviewed in^1^).

More recently, localized protein synthesis has been revealed in axons and presynaptic terminals^3,9^. Specifically, local protein synthesis is important for the response of the axonal growth cone to guidance cues^9^, and axonal translation of the t-SNARE protein SNAP25 was found to be necessary for the proper assembly of presynaptic terminals during development^10^. In adulthood, axonal protein synthesis plays a critical role in response to nerve injury^11^; mTOR is rapidly translated upon injury and regulates its own translation, as well as the levels of retrograde signaling proteins^11^. The role of presynaptic protein synthesis in plasticity and behavior is less well characterized, though it is necessary for branch-specific long-term facilitation in *Aplysia*^12,13^, and presynaptic protein synthesis is induced in mammalian primary neurons upon multiple plasticity-inducing stimuli^3^. More recent studies in the mammalian brain have found that long-term plasticity of GABA release^14^ and neurotransmitter release at the calyx of Held^15^ both involve presynaptic translation.

In order to further our understanding of how presynaptic protein synthesis regulates plasticity and behavior, it is critical to identify presynaptically localized transcripts. Recent studies identified the axonal transcriptome and translatome of cultured motor neurons^16^ and retinal ganglion cells^17^, respectively, as well as transcripts enriched in synaptosomes containing the vesicular glutamate transporter^3^; however, the full set of transcripts in the nervous system that are localized specifically to presynaptic compartments have yet to be described in any system. Furthermore, it is unknown if presynaptically localized transcripts contribute to complex behaviors.

We recently developed a technique to isolate and RNA-sequence specific tissues and neuronal subtypes in the nematode worm *C. elegans*^18^, revealing new regulators of neuron-specific phenotypes, such as axon regeneration and associative learning and memory. Here we describe how we have differentially labeled somatic, axonal, and presynaptic compartments of the adult *C. elegans* nervous system using a dual-fluorescent protein strategy. These differentially labeled compartments can be isolated by fluorescence activated cell sorting (FACS) and used to identify presynaptically-localized RNAs. We find that these “synapse-expressed” genes have predicted synaptic functions. We also use this technique to identify genes that are enriched in presynaptic compartments relative to somatic compartments of the same neuronal populations. We highlight the ability of this technique to rapidly identify novel and conserved presynaptic mRNAs, by determining that mammalian orthologs of synapse-enriched genes are known to function in synaptic and axonal regions. We also demonstrate the ability of this technique to identify novel, presynaptic mRNAs that contribute to neuronal functions: presynaptically-enriched *C. elegans* Pumilio and FBF (PUF) RNA-binding proteins, orthologs of mammalian *Pumilio*, are necessary for normal associative memory. Because the regulation of synaptic transmission and presynaptic function is highly conserved between *C. elegans* and mammals, this method allows for the rapid identification of synaptically-localized transcripts likely to function in higher organisms to regulate processes where local protein synthesis is required, such as repair and plasticity.

## Results

### Isolation of pan-neuronal presynaptic transcripts using a dual-fluorescent labeling strategy

**Figure 1:**
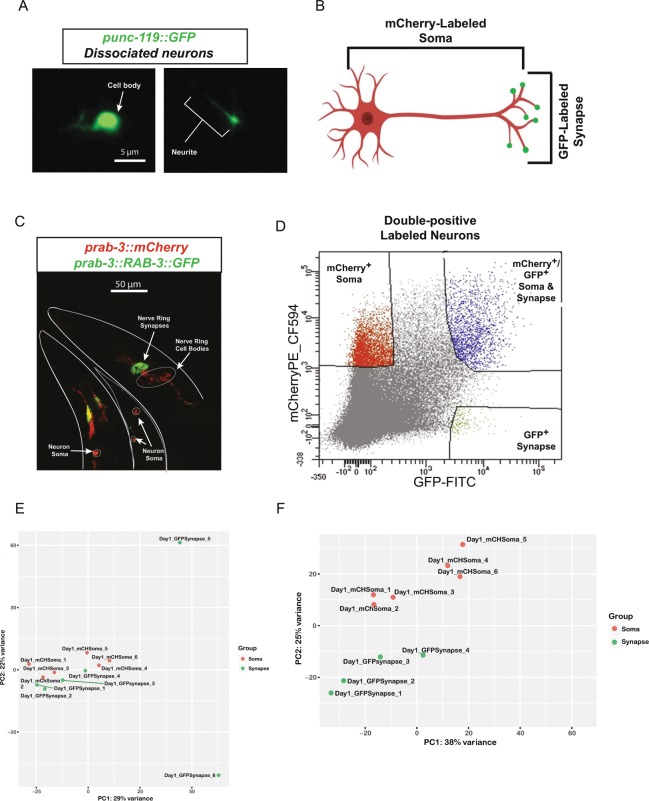
Isolation of presynaptic compartments for RNA-seq. (**A**) Neuronal cell isolation results in fragmentation of cells, where cell bodies (left) can be detected separately from neurites (right). (**B**) Schematic of the dual-fluorescent protein strategy for isolating synaptic and somatic compartments in *C. elegans*. (**C**) Representative confocal images of *rab-3p::mCherry; rab-3p::RAB-3::GFP* transgenic worms. Neurons in the head (outlined in white) and tail (outlined in gray) show distinct expression of fluorescent proteins. Cell bodies (outlined in white, arrows) express somatic mCherry (red), while nerve ring synapses exclusively express GFP (green, arrows. Colocalization of the two fluorescent proteins (yellow) is also evident. (**D**) FACS plot displaying ability to isolate synaptic (GFP+, green), somatic (mCherry+, red), and synaptic + somatic (GFP+/mCherry+, blue) compartments using adult neuron cell isolation method^18^. (**E,F**) Principle components analysis of six mCherry+ (red) somatic and GFP+ (green) synaptic RNA-seq samples performed as part of DESeq2 analysis. (**E**) Principle component analysis of four synaptic (green) and six somatic (red) RNA-seq samples after removal of outliers. Remaining samples cluster by subcompartment.

We previously developed a technique to identify the transcriptomes of adult *C. elegans* tissues^18,19^ that utilizes tissue-specific labeling by promoter-driven fluorescent proteins, outer cuticle breaking, size-specific filtering and sorting of cells by FACS, which can be used for RNA-isolation and transcriptome analysis by RNA-seq. Using this approach, we identified transcripts expressed in specific tissues and individual cell types and neurons^18^. We discovered that the enzymatic and mechanical dissociation steps of isolating neurons using this technique could result in their fragmentation; fluorescently labeled neurites were often observed during sample preparation (Fig. 1A). Because neurites and synapses contain their own mRNA, we devised a strategy to take advantage of this fragmentation and isolate specific neuronal sub-compartments, differentially labeling neurons with different fluorescent protein markers, which enabled us to simultaneously collect somatic and presynaptic regions from the same adult population of animals for transcriptomic analysis. Somatic regions were labeled with mCherry under the control of the promoter for the pan-neuronal Rab family GTPase *rab-3*, while a RAB-3::GFP translational fusion, which localizes to presynaptic regions and is a widely used synaptic marker^20^, was used to specifically label synapses (Fig. 1B). Differentially-localized fluorescent proteins were detectable by microscopy (Fig. 1C), and upon performing flow cytometry of isolated neurons from *pRab-3::mCherry;pRab-3::RAB-3::GFP* animals, GFP+ pre-synaptic regions were isolated independently of mCherry+ cell bodies, as well as double-positive (GFP+/mCherry+) events which likely contain intact axons (Fig. 1D). Each of these three isolated populations contained RNA that could be isolated and subjected to RNA-seq. We generated six biological replicates of these differentially-labeled fluorescent samples. Principle components analysis (PCA) revealed that all six mCherry+ soma samples clustered together, while two of the six GFP+ synaptic samples appeared to be outliers and were discarded from further analysis (Fig. 1E). The remaining samples clustered well by isolated subcompartment (Fig. 1F), and were used for further analysis (for alignment details, see Table S1).

### Isolation of presynaptic mRNAs reveals that presynaptically expressed genes characterize synaptic function

**Figure 2:**
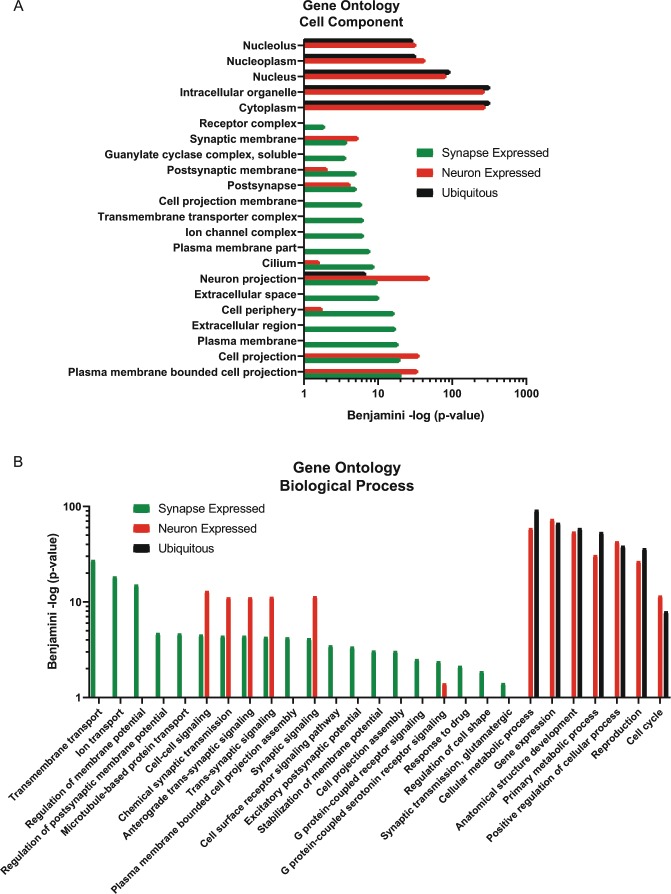
Isolation of presynaptic mRNAs reveals specialized neuronal functions. **(A,B**) GO Analysis was performed on “synapse-expressed” genes. Significant GO terms (padj < 0.05) highlight that synapse-expressed genes(green) are predicted to be specialized neuronal genes in terms of both localization (**A**) and function (**B**), enriched for membrane related terms and depleted for nuclear terms, unlike ubiquitous (black) and neuron-expressed genes (red).

In order to classify a gene as “expressed in synaptic compartments,” it had to have an average of 10 counts across the 4 remaining GFP+ synaptic samples. We additionally filtered out previously-identified ubiquitously-expressed genes, that are detected across all adult tissue samples^19^. Using these cutoffs, we identified 8,778 “synapse expressed” genes (Table S2). Comparison of GO terms of “synapse-expressed” genes (Table S2) to previously published neuron-expressed genes^19^ and ubiquitous genes^19^ revealed that “synapse-expressed” genes are predicted to have specialized neuronal functions that are synaptic in nature (Fig. 2A,B), suggesting that we were successful in isolating mRNAs localized to synaptic regions.

For example, cellular component GO terms shared between neuron-enriched and synapse-expressed gene sets included *neuron projection* and *synaptic membrane*, but some terms were exclusively synaptic, such as *receptor complex, ion channel complex*, and *plasma membrane* (Fig. 2A). Synapse-expressed genes were not predicted to function in other cellular components, such as the nucleus, which were highly significant in the neuron-expressed and ubiquitous gene lists (Fig. 2A), which suggests that our technique is depleted for somatic compartments, further validating our isolation of synaptic regions.

We also used GO analysis to examine the predicted functions of synapse-expressed genes. Functions such as *ion transport, regulation of membrane potential, microtubule based protein transport*, and *synaptic transmission, glutamatergic* (Fig. 2B) were exclusive to synapse-expressed genes. Shared terms with neuron-expressed genes included *synaptic signaling, chemical synaptic transmission*, and *cell-cell signaling*, but again terms that denoted nuclear functions such as *gene expression* were absent in synapse-expressed genes (Fig. 2B). These results suggest that we have identified genes that contribute to well-characterized aspects of synaptic function.

### Identification of synaptic differentially expressed genes (DEGs)

**Figure 3:**
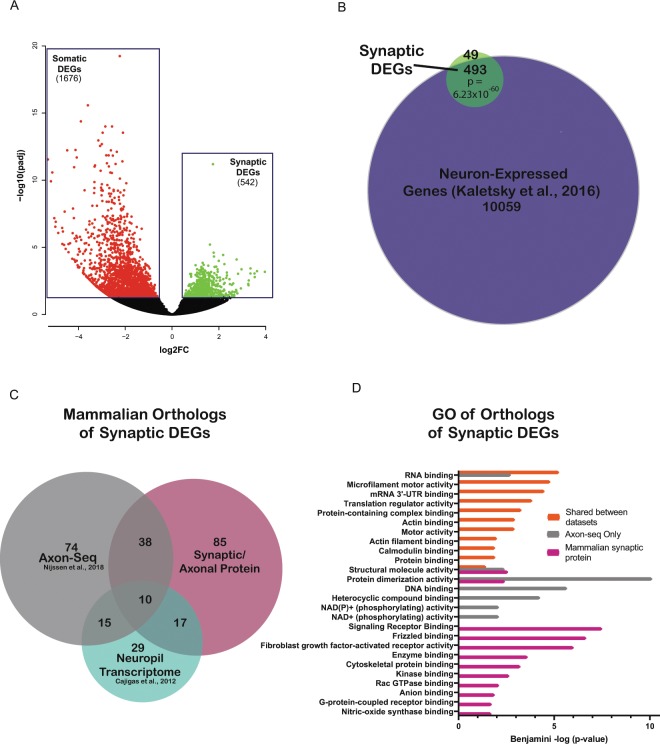
Characterization of Synaptic DEGs. (**A**) Volcano plot of Synaptic DEGs (Green) relative to Somatic (Red) samples. FDR for DEGs = 0.05 (**B**) Synaptic DEGs significantly overlap with previously identified adult-neuron expressed genes^18^ P-values: hypergeometric distributions (**C**) Mammalian orthologs of *C. elegans* synaptic DEGs function in synapses (for citations, see Table S4) or have been detected in previous synaptic neuropil^7^ (blue) and axonal transcriptomic^16^ (grey) datasets. (**D**) GO analysis of known conserved synaptic DEGs reveals synaptic and axonal functions (orange, grey, magenta), and an enrichment of RNA binding proteins and translational regulators (orange).

Because our method simultaneously isolates synaptic and somatic compartments from the same neuronal population (Fig. 1D), we could not only identify mRNAs that were present at the synapse, but we could also determine which mRNAs were most significantly enriched in presynaptic regions. To identify synaptically-enriched genes, we used DESeq2 for differential expression analysis^21^ to determine which transcripts were expressed at significantly higher levels (FDR < 0.05) in synaptic samples (GFP^+^, Fig. 1D) relative to somatic samples (mCherry^+^, Fig. 1D), revealing 542 synaptic DEGs (Fig. 3A, Table S3). These synaptic DEGs were significantly enriched (~91%, p = 6.23 × 10^−60^, hypergeometric test) for previously-identified adult neuronal genes^18^ (Fig. 3B), confirming that these DEGs were a subset of neuronal genes that are enriched in presynaptic regions.

### Synaptic DEGS have known synaptic and axonal functions in mammals

*C. elegans’* nervous system has a high degree of conservation with mammalian neurons. We were therefore interested in determining if our technique had identified synaptic mRNAs that were evolutionarily conserved. Using the OrthoList2 tool^22^, we found that 311 of the *C. elegans* synaptic DEGs have predicted mammalian orthologs (Table S4). Of those mammalian orthologs, 269 have been previously validated as axonal or synaptic: protein products of 150 genes were found to  either function in synapses and axons (Synaptic/Axonal Protein, Fig. 3C), while previous transcriptomic studies that captured subsets of axonal or synaptic regions identified orthologs of 137 and 71 synaptic DEGS, respectively (Axon-seq and Neuropil transcriptome, Fig. 3C^7,16^). Many orthologs were unique to each gene set (Fig. 3C); suggesting that our nervous-system wide profiling of the synaptic transcriptome has found new synaptically-localized transcripts in multiple neuron subtypes. GO analysis of orthologs unique to the “Synaptic/Axonal Protein” and “Axon-Seq” list (Grey and Magenta Circles, Fig. 3C) indicated that these genes were indeed synaptic in function (Fig. 3D): previously-identified mammalian synaptic proteins are involved in *signaling receptor binding*, including a number of molecules involved in Wnt/Frizzled signaling (*dsh-2/Dvl1-3, egl-20/Wnt16)* and fibroblast growth receptor signaling (*egl-15/Fgfr1-4), cytoskeletal protein binding* including several kinesins *(klc-1/Klc2, klp-15;klp-16/Kifc3)*, and genes that bind anions and kinases. Axon-seq-unique orthologs also included structural and cytoskeletetal-regulating molecules (Fig. 3D). Due to the relatively low number of orthologs (29, Fig. 3C) that are unique to the synaptic neuropil list, we were unable to detect GO term enrichment; however, a number or these genes are receptor and membrane-associated (*yop-1/Reep6*, *Y57E12A.1/Serinc2, flap-1/Lrrfip2)*, microtubule associated (*C14H10.2/Jakmip1;Jakmip2*), and intracellular signaling molecules (*Y105E8A.2/Arhgef2, skr-2/Skp1*). The large number of genes involved in signaling and cytoskeletal regulation present in synaptic DEGS suggests that they are important for the dynamic remodeling that occurs in axons and synapses following unique stimuli.

### Presynaptic mRNA isolation reveals that transcripts of translational regulators and RNA binding proteins are enriched in presynaptic regions

*C. elegans* synaptic DEGS orthologs shared between datasets may have an especially important role in regulating synaptic function. GO analysis of all genes shared between any two datasets (Axonal/synaptic protein, Axon-seq, and Synaptic Neuropil) revealed expected terms such as *actin binding, actin filament binding*, and *motor activity* (Fig. 3D). The most significantly enriched GO term for the shared orthologs was *RNA binding*, with *mRNA 3*′ *UTR binding* and *translation regulator activity* also represented in the shared data set (Fig. 3D). We determined which genes contributed to these GO terms. Translation regulators included five eukaryotic initiation factors (eIFs*, iff-1/eIF5A, ife-1/eIF4E, drr-2/eIF4H, ife-3/eIF4E, gcn-2/eIF2AK4*), two ribosomal subunits, and a eukaryotic elongation factor (*eef-1B.2/eEF1B2*). Multiple *C. elegans* orthologs of mammalian RNA binding proteins, including RNA Binding Motif protein 3/Cold inducible RNA binding protein (*rbm-3.1, rbm-3.2)* and Y-box binding protein 3 (*cey-2, cey-3*) were also present. Interestingly, the most significantly most numerous RNA binding proteins present in the synaptic DEGs were *pufs* (*puf-3, puf-5, puf-7, puf-8, puf-11)* which are orthologs of mammalian *Pumilio1 and Pumilio2 (Pum1/2)*.

### The PUM binding motif is enriched in 3′UTRs of presynaptic transcripts and DEGs

The presence of numerous *pufs* in the synaptic DEG set suggested that they might regulate synaptically-localized mRNAs. To examine this, we performed analysis of motif enrichment (AME) using the MEME Suite of motif-based sequence analysis tools^23^. This analysis revealed that several permutations of the PUMILIO binding motif (UGUACAK, UGUAMAK, UGUAYAK) were significantly enriched in both the “synapse expressed” and synaptic DEG lists (Table S5). These results suggest that PUFs may indeed be important regulators of synaptic mRNA localization and function, and we therefore chose the members of this family of RNA binding proteins for further characterization.

### PUMILIOs identified by presynaptic mRNA isolation have neuronal and axonal mRNA localization, and regulate behavior

**Figure 4:**
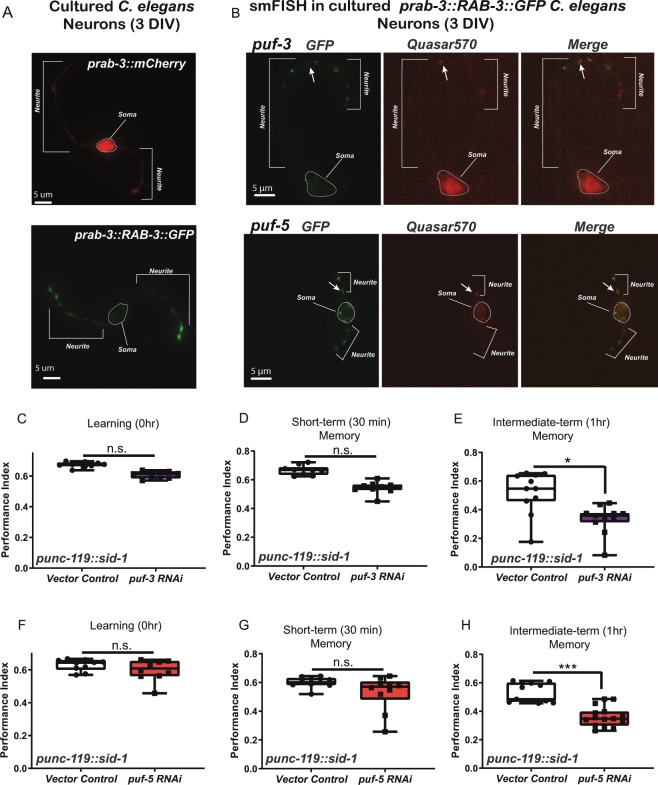
*puf* mRNAs are neuronally and axonally localized, and PUFs regulate associative memory. (**A**) Isolated *C. elegans* neurons 3 days *in vitro* (DIV), from animals expressing either somatic (*prab-3::mCherry)* and synaptic (*prab-3::RAB-3::GFP)* markers. Soma are circled and labeled, and neurites are indicated on the images. (**B**). smFISH using Quasar570-labeled probes against individual *puf* mRNAs in isolated *prab-3::RAB-3::GFP* neurons. Soma are circled and labeled, and neurites are indicated on the images. (**C–E**) *puf-3* is not required for learning (**C**) or short-term memory (**D**), but is necessary for intermediate-term memory (**E**). (**F–H**) *puf-5* is not required for learning (**F**) or short-term memory (**G**), but is necessary for intermediate-term memory (**H**). Mean ± SEM. n ≥ 8–10 per RNAi treatment. *p < 0.05, ***p < 0.001. Mean ± SEM. n ≥ 8–10 per genotype.

Although mammalian PUM1/2 are expressed in neurons, *C. elegans* PUFs have primarily been characterized in the germline, where they regulate processes such as germ cell development, germline proliferation, and oocyte maturation^24^. To confirm our sequencing data, we examined whether *puf* mRNAs were present in neurons and co-localized with the presynaptic marker RAB-3::GFP. We cultured isolated *C. elegans* neurons from transgenic worms expressing *rab-3p::mCherry* (soma label, Fig. 4A) and *rab-3p::RAB-3::GFP* (synaptic label, Fig. 4A). mCherry signal was primarily detected in the nucleus and soma of cultured neurons, while RAB-3::GFP was detected in distinct puncta along neuronal projections (Fig. 4A). To visualize mRNAs, we performed single molecule fluorescent *in situ* hybridization (smFISH^25^) on cultured *C. elegans* neurons using probes designed against individual *puf* mRNAs. We confirmed that synaptically-enriched *puf* mRNAs (*puf-3, puf-5*, Fig. 4B, *puf-7, puf-8, puf-11*, Fig. S1) identified by RNA-seq are indeed expressed in neurons (Fig. 4B), and that mRNA puncta for individual *puf* mRNAs co-localize with synaptic RAB-3::GFP. These results suggest that specific *puf* mRNAs are transported to pre-synaptic regions, presumably in preparation for translation.

We were also interested in determining if our technique could identify genes that could regulate neuronal function. What processes might axonal *pufs* regulate? We previously found that molecules that regulate presynaptic transport and transmission are important for learning and associative memory formation^18,26–28^. Furthermore, *pumilio* is an important regulator of long-term memory in *Drosophila*^29^, and mammalian PUM1/2 regulate dendrite morphogenesis, synaptic function, neuronal excitability, and hippocampal neurogenesis^30–32^. We therefore assessed the role of presynaptic *pufs* in positive olfactory associative memory, in which worms form a positive association with the neutral odorant butanone after pairing with food. A single food-butanone pairing results in a translation-dependent, intermediate-term memory one hour post-training that is forgotten in an active, translation-dependent manner by two hours post-training^33,34^. Adult-specific, RNAi-mediated knockdown of *puf-3* and *puf-5* in neuronal-RNAi-sensitive animals resulted in selective intermediate-term memory deficits (Fig. 4E,H), while translation-independent learning and short-term memory were unaffected by knockdown (Fig. 4C,D,F,G). These results suggested that *puf-3* and *puf-5* are essential memory promoting factors, which is in agreement with previous findings in *Drosophila*^29^.

## Discussion

Here we describe a rapid, simple approach for the isolation of synaptic mRNAs. There are several major advantages to our method. First, by using *C. elegans*, we can rapidly assess the adult synaptic transcriptome due to their short developmental timecourse (Day 1 of adulthood occurs three days after hatching) relative to other organisms. Second, this technique takes advantage of the many genetic tools available in *C. elegans* to differentially label and isolate the synaptic and somatic compartments. Here we used pan-neuronal somatic and synaptic markers to define the adult, nervous-system-wide presynaptic transcriptome for the first time in any system. We find that transcripts localized to synaptic regions reflect specialized neuronal functions that are expected in genes that would regulate plasticity, demonstrating that this technique is successful in enriching synaptic transcripts independently of somatic regions.

Another major advantage of this method is that it enables the simultaneous isolation of synaptic and somatic mRNAs from the same neuronal population, which allows not only for detection of genes expressed in synaptic regions, but also differential expression analysis to determine which genes are enriched, and potentially depleted, in synaptic compartments. This technique will be particularly useful in future studies that compare the synaptic and somatic transcriptomes of different genotypes or treatment conditions. Here, we used the technique to find pan-neuronal synaptic DEGs, which are the most enriched in synaptic relative to somatic regions. These DEGs include receptors, molecular motors, and regulators of cytoskeletal remodeling and axon outgrowth, all of which are critical components of synaptic remodeling that occurs during plasticity.

Moreover, our presynaptic mRNA isolation will enable the identification of new mechanisms that regulate neuronal function. For example, we examined the role of a subset of the synaptic DEGS, the *puf* RNA binding proteins, orthologs of mammalian PUM1/2, in complex behavior. We confirmed that these *puf* mRNAs are indeed neuronal and co-localize with synaptic markers. Another *C. elegans pumilio* ortholog, *fbf-1*, has been previously shown to function in neurons and regulate behavior^33,35^, but this is the first time that *puf* members of the *pumilio* gene family members have been found *C. elegans* neurons. It is not entirely unexpected that *pumilios are* in axonal regions. FMRP, which is known to interact with PUM2^31^, is localized in axons during synaptogenesis^36^, so it is likely that other FMRP partners also exhibit axonal localization in higher organisms. Moreover, mammalian PUM2 was recently found to play a role in regulating axonal localization of transcripts in the developing nervous system via restricting mRNAs to the soma^37^. Our results suggest that PUFs play an important role within the axonal and presynaptic regions in adult animals. In the future, it will be interesting to further study the regulatory role of PUMs/PUFs across development and lifespan.

We find that synaptically-localized *pufs* are necessary for normal associative memory formation. In addition to the *pufs*, we also found an unexpectedly large number of translational regulators and RNA-binding proteins in the list of synaptic DEGs. One possibility for this abundance is to enable stimulus-specific responses in neurons: regulating the expression of translational regulators can provide an additional layer of translational control. It will be interesting to determine what aspects of synaptic function these proteins contribute to in the future.

Overall, our data reveal the presynaptic transcriptome, and we demonstrate that presynaptic transcripts contribute to associative behaviors. Because many of these transcripts we identified have conserved functions in mammals, these findings set the framework for future studies for understanding the role that these presynaptic proteins play in plasticity, behavior and repair.

## Methods

### Experimental model and subject details

#### C. elegans genetics

All strains were maintained at 20 °C on plates made from standard nematode growth medium (NGM: 3 g/L NaCl, 2.5 g/L Bacto-peptone, 17 g/L Bacto-agar in distilled water, with 1 mL/L cholesterol (5 mg/mL in ethanol), 1 mL/L 1 M CaCl_2_, 1 mL/L 1 M MgSO_4_, and 25 mL/L 1 M potassium phosphate buffer (pH 6.0) added to molten agar after autoclaving^38^; or high growth medium (HGM: NGM recipe modified as follows: 20 g/L Bacto-peptone, 30 g/L Bacto-agar, and 4 mL/L cholesterol (5 mg/mL in ethanol); all other components same as NGM), with OP50 E. coli as the food source. Experiments that did not involve RNAi treatments were performed using NGM and HGM plates seeded with OP50 E. coli for *ad libitum* feeding^38^; for RNAi experiments, the standard HGM molten agar was supplemented with 1 mL/L 1 M IPTG (isopropyl β-d-1-thiogalactopyranoside) and 1 mL/L 100 mg/mL carbenicillin, and plates were seeded with HT115 E. coli for *ad libitum* feeding. Hypochlorite-synchronization to developmentally synchronize experimental animals was performed by collecting eggs from gravid hermaphrodites via exposure to an alkaline-bleach solution (*e.g*., 8.0 mL water, 0.5 mL 5 N KOH, 1.5 mL sodium hypochlorite), followed by repeated washing of collected eggs in M9 buffer (6 g/L Na_2_HPO_4_, 3 g/L KH_2_PO_4_, 5 g/L NaCl and 1 mL/L 1 M MgSO_4_ in distilled water^38^). For RNAi experiments, animals were transferred at the L4 larval stage onto HGM-RNAi plates until Day 2 of adulthood, when the animals were subjected to behavioral testing.

Strains. Wild-type: (N2 Bristol); Transgenic strains: NM2415 (*lin-15B(n765); jsIs68[Prab-3::GFP::rab-3* + *lin-15(+)]*), LC108 (*vIs69 [pCFJ90(Pmyo-2::mCherry* + *Punc-119::sid-1)]*), OH441(*punc-119::GFP) were provided by the Caenorhabditis Genetics Center (CGC)*. The transgenic strain CQ574 (*lin-15B(n765); jsIs682 [Prab-3::GFP::rab-3* + *lin-15(+)]; wqIs3 [Prab3::mCherry*]) was generated by UV integration^39^ of NM2415 (*lin-15B(n765); jsIs68[Prab-3::GFP::rab-3* + *lin-15(+)]*) animals microinjected with a *Prab3::mCherry* transgenic construct, followed by 3 rounds of outcrossing with wild-type (N2 Bristol) worms.

### Method details

#### Adult cell isolation

Adult cell isolation was performed as described previously^18^. Synchronized day 1 adult CQ574 (*lin-15B(n765); jsIs682 [Prab-3::GFP::rab-3* + *lin-15(+)]; wqIs3 [Prab3::mCherry*]) worms washed with M9 buffer to remove excess bacteria. The pellet (~250 µl) was washed with 500 µl lysis buffer (200 mM DTT, 0.25% SDS, 20 mM Hepes pH 8.0, 3% sucrose) and resuspended in 1000 µl lysis buffer. Worms were incubated in lysis buffer with gentle rocking for 6.5 minutes at room temperature. The pellet was washed 6× with M9 and resuspended in 20 mg/ml pronase from *Streptomyces griseus* (Sigma-Aldrich). Worms were incubated at room temperature (<20 minutes) with periodic mechanical disruption by pipetting every 2 min. When most worm bodies were dissociated, leaving only small debris and eggs, ice-cold  osmo-balanced Leibovitz's L-15 buffer containing 2% fetal bovine serum (Gibco) was added. RNA from FACS-sorted neurons was prepared for RNA-seq and subsequent analysis (see FACS isolation and RNA seq Analysis for more details).

#### FACS isolation of dissociated cells

Cells were briefly subjected to SDS-DTT treatment, proteolysis, mechanical disruption, cell filtering, as described in Adult cell isolation (above). Neuron cell suspensions were passed over a 5 μm syringe filter (Millipore). The filtered cells were diluted in osmo-balanced Leibovitz’s L-15/2% FBS and sorted using a FACS Aria IIIw/ DiVa (BD Biosciences; 488 nm excitation for GFP detection, 568 nm excitation for mCherry detection). Gates for detection were set by comparison to non-fluorescent N2 cell suspensions prepared on the same day from a population of worms synchronized alongside the experimental samples. Positive fluorescent events were sorted directly into Eppendorf tubes containing Trizol LS for subsequent RNA extraction. For each sample, approximately 30,000–130,000 GFP or mCherry positive events were collected, yielding 1–10 ng total RNA.

#### RNA isolation, amplification, library preparation, and sequencing

RNA was isolated from FACS-sorted samples as previously described^18,19^. Briefly, RNA was extracted using standard Trizol/chloroform/isopropanol method, DNase digested, and cleaned using Qiagen RNEasy Minelute columns. Agilent Bioanalyzer RNA Pico chips were used to assess quality and quantity of isolated RNA. RNA sequencing libraries were prepared directly from quality assessed RNA using the SMARTer Stranded Total RNA kit v2-Pico input mammalian, as per manufacturer suggested practices. The resultant sequencing libraries were then submitted for sequencing on the Illumina HiSeq 2000 platform. ~75–190 million reads (average of 128,910,533 reads) were obtained for each sample and mapped to the *C*. *elegans* genome. Raw sequencing reads are available at NCBI Bioproject: PRJNA559377.

#### Microscopy

Imaging of day 1 CQ574 (*lin-15B(n765); jsIs682 [Prab-3::GFP::rab-3* + *lin-15(+)]; wqIs3 [Prab3::mCherry*]) adults was performed on a Nikon A1 confocal microscope at 60× magnification, and *z* stacks were processed in Nikon NIS elements software. For imaging of smFISH samples, Z-stack multi-channel (DIC, TRITC, GFP) of isolated neurons were imaged every 0.2 µm at 100X magnification on a Nikon Eclipse Ti inverted microscope; Maximum Intensity Projections and 3D reconstructions of neurons were built with Nikon *NIS-Elements*.

#### *C. elegans* neuronal cell isolation and culture

Isolation and culture of neurons was performed as previously described^40^, with modifications. Animals were synchronized by hypochlorite treatment and grown on OP50-seeded NGM or HGM plates until the L4 larval stage. One hour prior to cell isolation, animals were allowed to incubate in M9 buffer to clear the gut of bacteria. After one hour, animals were incubated in 200–300 µl freshly thawed sterile SDS-DTT solution (200 mM DTT, 0.25% SDS, 20 mM HEPES, pH 8.0, 3% sucrose, stored at −20 °C) for 4 min at room temperature. The animals were washed 3–5 times in M9 buffer and pelleted by centrifugation in a tabletop centrifuge. Animals were then digested in 15 mg/ml pronase for 20–25 min and subjected to mechanical disruption by frequent pipetting. Pronase digestion was stopped by adding 900 µl L-15 medium (Invitrogen, Carlsbad, CA) supplemented with 10% fetal bovine serum (Invitrogen, Carlsbad, CA), 50 U/ml penicillin, and 50 µg/ml streptomycin (Sigma-Aldrich, St. Louis, MO) and adjusted to 340 mOsm. Cells were pelleted by centrifugation at 10,000 rpm for 5 min at 4 °C, and washed 2 times with L-15/FBS. The pellet was resuspended with 1 ml L-15/FBS and settled on ice for 30 min. The top 800 µl cell suspension devoid of large worm debris was transferred to a new tube and pelleted by centrifugation at 10,000 rpm for 5 min at 4 °C. Cell pellets were resuspended in fresh L-15/FBS. 40 to 50 µl of cell suspension was plated onto the center an acid-washed coverslip coated with 0.5 mg/ml peanut lectin (Sigma-Aldrich, St. Louis, MO). Cells were allowed to adhere overnight in a 20 °C incubator without CO_2_ in Snapware plastic containers and humidified with moist paper towels. 24 hours later, debris and unbound cells were washed off with L-15, and 1 ml of fresh L-15/FBS was added to the coverslips. After 3 days in culture, smFISH was performed.

#### Single molecule fluorescence *in situ* hybridization

Custom Stellaris® FISH Probes were designed against *puf-3, puf-5, puf-7, puf-8*, and *puf-11* by utilizing the Stellaris® RNA FISH Probe Designer (Biosearch Technologies, Inc., Petaluma, CA) available online at www.biosearchtech.com/ stellarisdesigner (version 4.2). The isolated neurons from *C. elegans* strain NM2415 (*lin-15B(n765); jsIs68[Prab-3::GFP::rab-3* + *lin-15(+)]*) were hybridized with individual *puf* Stellaris RNA FISH Probe sets labeled with Quasar 570 (Biosearch Technologies, Inc.), following the manufacturer’s instructions available online at www.biosearchtech.com/stellarisprotocols. Briefly, cells were fixed with a paraformaldehyde fixation buffer (3.7% paraformaldehyde in 1X PBS) for 10 minutes at room temperature, washed twice with 1X PBS, and permeabilized for at least 1 hour with 70% EtOH. After permeabilization, cells were washed with Wash Buffer A (Biosearch Technologies, Inc.) containing 10% deionized formamide (Sigma Aldrich), and hybridized overnight at 37 °C with Stellaris RNA FISH Probe sets at a final concentration of 250 nM in Hybridization buffer (Biosearch Technologies, Inc.) containing 10% deionized formamide (Sigma Aldrich). After hybridization, cells are washed twice at 37 °C with Wash Buffer A (Biosearch Technologies, Inc.) containing 10% deionized formamide (Sigma Aldrich), followed by a 2–5 minute wash at room temperature with Wash Buffer B (Biosearch Technologies, Inc.). Coverslips were then applied to slides and imaged (see *Microscopy* section).

#### Behavioral assays

Vector Control or RNAi-treated animals were trained and tested for or short/intermediate term memory as previously described^34^. Briefly, synchronized day day 2 adult worms for RNAi treated animals were washed from HGM plates with M9 buffer, allowed to settle by gravity, and washed again with M9 buffer. After washing, the animals are starved for 1 hr in M9 buffer. For 1 food-butanone pairing, worms were then transferred to 10 cm NGM conditioning plates (seeded with OP50 *E. coli* bacteria and with 6 μl 10% 2-butanone (Acros Organics) in ethanol on the lid) for 1 hr. After conditioning, the trained population of worms were tested for chemotaxis to 10% butanone vs. an ethanol control either immediately (0 hr) or after being transferred to 10 cm NGM plates with fresh OP50 for specified intervals before testing (30 mins-2 hrs), using standard, previously described chemotaxis assay conditions^41^.

Chemotaxis indices were calculated as follows: **(#worms**_**Butanone**_ − **#worm**_**Ethanol**_**)/(Total #worms)**. Performance index is the change in chemotaxis index following training relative to the naïve chemotaxis index. The calculation for Performance Index is: **Chemotaxis Index**_**Trained**_ − **Chemotaxis Index**_**Naive**_.

#### Motif analysis

Motif discovery and analysis was performed using the AME tool^42^ of the MEME Suite^23^. Full length annotated 3′ UTRs were downloaded using WormBase Parasite^43^. Duplicates were removed and provided to the AME tool accordingly, and motifs were tested for enrichment using the tool-provided RNA-binding motif database^44^.

### Quantification and statistical analysis

#### RNA-seq data analysis

FASTQC was used to inspect the quality scores of the raw sequence data, and to look for biases. Reads were mapped to the *C*. *elegans* genome (WormBase 245) using STAR with WormBaseID gene model annotations (using default parameters). Count matrices were generated for the number of reads overlapping with the gene body of protein coding genes using htseqCounts. DESeq2 was used for differential expression analysis and the principal components analysis. Genes at FDR = 0.05 were considered significantly differentially expressed.

#### Gene ontology analysis

Hypergeometric tests of Gene Ontology terms were performed on tissue-enriched gene lists using g:Profiler (https://biit.cs.ut.ee/gprofiler/gost); GO terms reported are a significance of *q-value* < 0.05 unless otherwise noted.

#### Behavioral assay analysis

For the comparison of performance indices between two RNAi treatments (i.e. Vector control RNAi and *puf-3* RNAi), two-tailed unpaired Student’s t-tests with Welch’s corrections were used. Experiments were repeated on separate days with separate populations, to confirm that results were reproducible. Prism 8 software was used for all statistical analyses. Additional statistical details of experiments, including sample size (with n representing the number of chemotaxis assays performed for behavior, and number of cells imaged for microscopy), can be found in the figure legends.

## Supplementary information

Supplementary Information.

Dataset 1.

Dataset 2.

Dataset 3.

Dataset 4.

Dataset5.
