# Supplementary material for: Nervous system-wide profiling of presynaptic mRNAs reveals regulators of associative memory
Source: Sci Rep. 2019 Dec 30;9:20314. doi: 10.1038/s41598-019-56908-8 (PMC6937282; doi:10.1038/s41598-019-56908-8)
Supplement: Supplementary file 1 — Supplementary Information. [file 41598_2019_56908_MOESM1_ESM.pdf]

Nervous system-wide profiling of presynaptic mRNAs reveals regulators of associative memory

Rachel N Arey[1,2], Rachel Kaletsky[2], and Coleen T Murphy[2]*

Supplementary Information.

Supplementary Figure S1. Other *puf* mRNAs are neuronally and axonally localized A-C). smFISH using Quasar570-labeled probes against individual *puf* mRNAs (*puf-7, puf-8, puf-11*) in isolated *prab-3::RAB-3::GFP* neurons. Soma are circled and labeled, and neurites are indicated on the images.

Table S1. Alignment summary of Sequencing Data

Table S2. List of Synapse expressed genes and Gene Ontology for synapse expressed genes

Table S3. Results of DESeq2 analysis - Synaptic and Somatic DEGs

Table S4. Lists of mammalian orthologs of synaptic DEGs

Table S5. Motif analysis summary of synaptic mRNA 3'UTRs

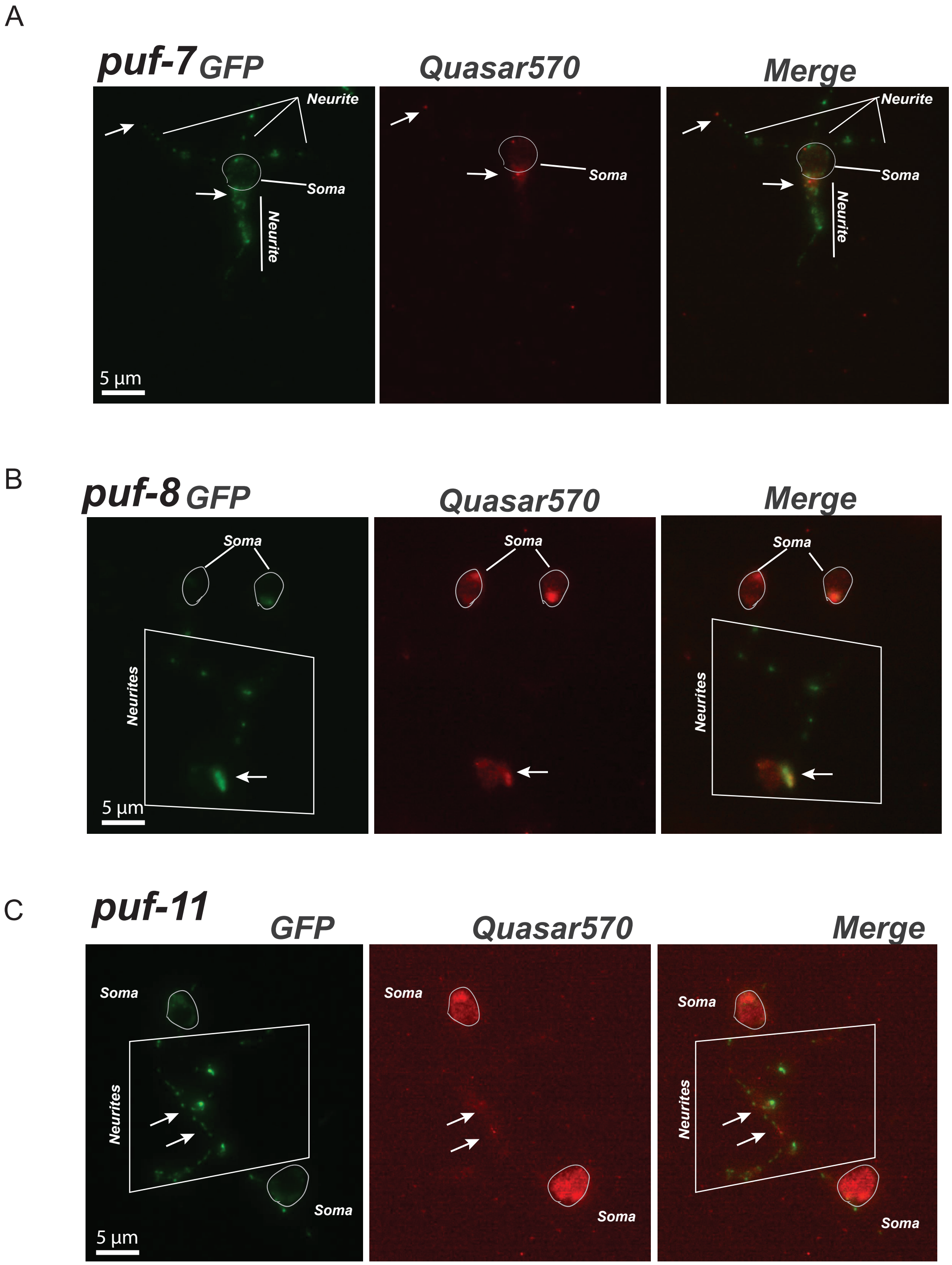
A
puf-7 GFP
Quasar570
Merge
Neurite
Soma
Neurite
5 µm
Neurite
Soma
Soma
Neurite
B
puf-8 GFP
Quasar570
Merge
Soma
Neurites
5 µm
Soma
Soma
Neurites
C
puf-11
GFP
Quasar570
Merge
Soma
Neurites
Soma
5 µm
Soma
Soma
Soma
Neurites
Soma

Figure S1
